# Fast Realistic MRI Simulations Based on Generalized Multi-Pool Exchange Tissue Model

**DOI:** 10.1109/TMI.2016.2620961

**Published:** 2016-10-25

**Authors:** Fang Liu, Julia V. Velikina, Walter F. Block, Richard Kijowski, Alexey A. Samsonov

**Affiliations:** Department of Radiology, University of Wisconsin at Madison, Madison, Wisconsin, USA; Department of Medical Physics, University of Wisconsin at Madison, Madison, Wisconsin, USA; Department of Medical Physics and Biomedical Engineering, University of Wisconsin at Madison, Madison, Wisconsin, USA; Department of Radiology, University of Wisconsin at Madison, Madison, Wisconsin, USA; Department of Radiology, University of Wisconsin at Madison, Madison, Wisconsin, USA

**Keywords:** simulation, CEST, magnetization transfer, relaxometry, graphical processing unit (GPU)

## Abstract

We present MRiLab, a new comprehensive simulator for large-scale realistic MRI simulations on a regular PC equipped with a modern graphical processing unit (GPU). MRiLab combines realistic tissue modeling with numerical virtualization of an MRI system and scanning experiment to enable assessment of a broad range of MRI approaches including advanced quantitative MRI methods inferring microstructure on a sub-voxel level. A flexibl representation of tissue microstructure is achieved in MRiLab by employing the generalized tissue model with multiple exchanging water and macromolecular proton pools rather than a system of independent proton isochromats typically used in previous simulators. The computational power needed for simulation of the biologically relevant tissue models in large 3D objects is gained using parallelized execution on GPU. Three simulated and one actual MRI experiments were performed to demonstrate the ability of the new simulator to accommodate a wide variety of voxel composition scenarios and demonstrate detrimental effects of simplifie treatment of tissue micro-organization adapted in previous simulators. GPU execution allowed ∼200× improvement in computational speed over standard CPU. As a cross-platform, open-source, extensible environment for customizing virtual MRI experiments, MRiLab streamlines the development of new MRI methods, especially those aiming to infer quantitatively tissue composition and microstructure.

## I. Introduction

Simulations constitute an essential part of the practice of magnetic resonance imaging (MRI) development as they allow for rapid prototyping and evaluation of MRI techniques in controlled conditions. Initially, analytical signal expressions based on simplifie descriptions of MRI processes for proton isochromats were commonly used for pulse sequence optimization and image contrast manipulation. Over the years, increasing complexity of MRI systems, emergence of novel acquisition and reconstruction methods, and exploration of advanced MRI contrast mechanisms necessitated more realistic MRI simulations based on numerical modeling [1, 2]. In turn, this stimulated development of dedicated software solutions that take advantage of growing availability of high-performance computing to increase fidelit of MRI simulations. The existing simulators comprise largely distinct sets of functionalities including basic MRI simulations [3], simulations in the presence of various imaging system imperfections [4-6], and evaluation of object-fiel interactions for optimization of specifi absorption rate (SAR), and multichannel transmission [7]. Several simulators feature graphical development interface for pulse sequence design [5, 8-10] and MRI technique prototyping [11]. Overall, the developed software solutions have contributed to a notable progress toward more accurate simulations of MRI hardware and imaging processes in acceptable time, though several important limitations still exist.

The major limitation of existing MRI simulators is the use of simplifie tissue representations based on a model where all protons reside in a single compartment instead of a more realistic biological model where protons interact in multiple compartments. As a result, even for basic MRI pulse sequences, the MRI signal and contrast in tissues cannot be fully described by the single compartment models. Instead, more sophisticated tissue models with multiple exchanging proton pools are generally required for adequate tissue representation [12]. The multi-pool modeling becomes especially important for advanced MRI techniques that move beyond pathology visualization and aim to characterize tissue composition, microenvironment, and microstructure in a quantitative fashion [13]. Examples of these approaches include quantitative magnetization transfer (MT) imaging (qMTI) [14-17], multi-component spin-lattice (*T*_1_) and spin-spin (*T*_2_) relaxometry [18-20], and chemical-exchange saturation-transfer (CEST) techniques [21]. Typically, these methods acquire several MR images with modulated contrast and utilize them to create quantitative or semi-quantitative parametric maps that characterize the tissue compartments. These parametric maps can often provide more biological or clinical information than conventional anatomical MRI images or basic quantitative MRI methods such as single-component *T*_1_/*T*_2_ maps. For example, multi-compartmental modeling of fat and water provides a quantitative indicators of fatty liver infiltratio [22], while quantitative dynamic contrast-enhanced MRI characterizes permeability changes often present in cancerous lesions [23]. The multi-pool representations can also be applied to model and correct macroscopic effects such as partial voluming of cerebrospinal flui (CSF) and neural tissues in brain imaging [24] or synovial flui and cartilage in knee imaging [25]. While specialized software [26] is available for general analysis of some models, there is a lack of tools for full-scale MRI simulations with generalized models. Hence, realistic MRI simulations with such models can provide valuable means to facilitate development, evaluation, and understanding of quantitative MRI approaches.

Excessive computational burden of full-scale three-dimensional (3D) MRI simulations is already a pressing need in the area of MR simulations today. Extra computational load associated with the desired multi-pool modeling is expected to further exacerbate this issue. Currently, the most commonly used approach to address the high computational burden is to parallelize computations on computer clusters [4-6, 27] which are expensive and not widely available.

To meet the need to simulate more biologically relevant tissue models with realistic computational loads, we present a comprehensive MRI simulator, MRiLab, equipped with the generalized multi-pool exchange model for accurate MRI simulations. Inspired by an initial promise of Graphical Processing Units (GPU) to accelerate MRI simulations in a relatively inexpensive manner [28], we hypothesize that the computational complexity associated with the use of the generalized tissue model and realistic digital objects may be efficientl addressed by the GPU programming to allow simulating the complex phenomena on a personal computer (PC). To demonstrate the importance of advanced tissue modeling, we apply the new simulator to assess several quantitative MRI methods. Additionally, we evaluate the effects of simplifie treatment of several such techniques by single-pool-based simulations. Finally, we utilize multi-pool modeling capabilities of MRiLab to simulate effects of fat-water interference in macromolecular-rich tissues and validate them in a physical phantom. The MRiLab software is available at http://mrilab.sourceforge.net/ for free open-source access.

## II. Theory

### A. Generalized Multi-Pool Exchange Model

**Fig. 1:**
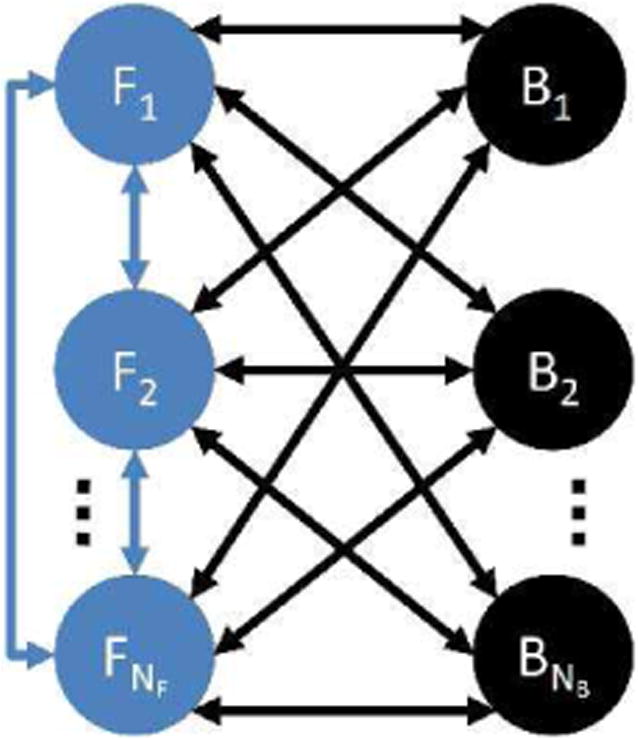
Generalized multi-pool exchange model. The tissue is represented by several free (*F*) and bound (*B*) proton pools undergoing the magnetization exchange.

Realistic modeling of MRI signal from a given volume element (voxel) requires taking into account multiple sources of protons with measurable magnetization and their interaction with protons with non-measureable (rapidly decaying) magnetization within a particular tissue type, as well as presence of several tissue types within the voxel. To accommodate the wide variety of the voxel composition scenarios, we propose to employ a generalized multi-pool exchange model shown in Fig. 1. The model consists of *N*_F_ free proton pools, all interconnected by the magnetization exchange pathways, and *N*_B_ bound proton pools exchanging with the free proton pools. The free proton pools represent compartments with measurable transverse magnetization (e.g., water, fat, solute proton exchange compounds), while the bound proton pools are used to model semi-solid tissue macromolecular content non-visible on standard MRI (e.g., myelin, muscle fibers collagen). A particular configuratio of the generalized model (i.e., number of the pools, their type, and exchange pathways between them) can be chosen along with its parameters (relative fractions of the proton pools, *T*_1_/*T*_2_ relaxation times, chemical shift spectra, and exchange rates) to represent a given tissue type.

The response of the multi-pool spin system to the sequence of radiofrequency (RF) pulses and imaging gradients (i.e., MRI pulse sequence) can be described using the finit differential Bloch-McConnell equations in the rotating frame [29] for free proton pools, and MT saturation formalism [30] for bound proton pools. The full system of the equations can be written as:

(1) $$\frac{d{\vec{M}}_{i}}{dt}=\gamma ({\vec{M}}_{i}\times {\vec{B}}_{i})-(\begin{array}{c} M_{x,i}/T_{2,i}-(\sum_{j\in F,j\neq i}K_{i,j}M_{x,i}-\sum_{j\in F,j\neq i}K_{j,i}M_{x,j})\\ M_{y,i}/T_{2,i}-(\sum_{j\in F,j\neq i}K_{i,j}M_{y,i}-\sum_{j\in F,j\neq i}K_{j,i}M_{y,j})\\ \frac{M_{z,i}-M_{0,i}}{T_{1,i}}-(\sum_{j\in F,j\neq i}K_{i,j}M_{z,i}-\sum_{j\in F,j\neq i}K_{j,i}M_{z,j}+\sum_{j\in B}K_{i,j}M_{z,j}-\sum_{j\in B}K_{j,i}M_{Z,j})\\ i=1,\ldots ,N_{F} \end{array})$$

(2) $$\frac{dM_{z,l}}{dt}=\frac{M_{z,l}-M_{0,l}}{T_{1,l}}-W(\Omega ,T_{2,l};t)M_{z,l}-\sum_{j\in F}K_{l,j}M_{z,l}+\sum_{j\in F}K_{j,l}M_{z,j}\;l=1,\ldots ,N_{B}$$

Here, the *i*^th^ free and *l*^th^ bound spin pools are each characterized by the equilibrium magnetization *M*_0,_*_i_* and *M*_0,*l*_, and by the magnetization vectors *M⃑_i_* and *M⃑_i_* = [*M_x,i_*, *M_y,i_*, *M_z,i_*], respectively. *M⃑_l_* = [0, 0, *M_z,l_*] denotes an effective magnetic fiel experienced by the *i*^th^ free spin pool, *γ* is the gyromagnetic ratio, and *K_i,j_* is the rate of magnetization exchange from *i*^th^ to *j*^th^ pools. Next, *W* stands for the time-dependent saturation rate of a bound proton pool:

(3) $$W(\Omega ,T_{2},t)=\pi {\left(\gamma |{\vec{B}}_{1\text{eff}}(t)|\right)}^{2}g(\Omega ,T_{2})$$

Here, *B⃑*_1eff_ is an effective transmit fiel in the transverse plane, **Ω** is RF offset frequency, *g*(**Ω**, *T*_2_) is macromolecular proton saturation line given in biological tissues and phantom media (e.g., agar, gelatin) by a super-Lorentzian

(4) $$g(\Omega ,T_{2})=\int_{0}^{1}\sqrt{\frac{2}{\pi }}\frac{T_{2}}{\left|3u^{2}-1\right|}e^{\left[-2{\left(\frac{2\pi \Omega T_{2}}{3u^{2}-1}\right)}^{2}\right]}du$$

and a Gaussian,

(5) $$g(\Omega ,T_{2})=\sqrt{\frac{2}{\pi }}T_{2}e^{-\frac{{\left(2\pi \Omega T_{2}\right)}^{2}}{2}}$$

respectively [31].

We construct the terms *B⃑_i_* in Eq. (1) to describe the applied magnetic fields macroscopic/microscopic fiel variations, off-resonance saturation, and chemical shifts (CS) of individual pools. The terms are specifie on a per-pool basis and composed of multiple sub-field as follows:

(6) $${\vec{B}}_{i}(\vec{r},t)={\vec{B}}_{1\text{eff}}(\vec{r},t)-\frac{\Omega (t)}{\lambda }\vec{Z}+(\vec{r},t)\vec{Z}+\Delta B_{\text{ma}}(\vec{r},t)\vec{Z}+C_{i}(\vec{r})\vec{Z}+\delta B_{\text{mi}}(\vec{r})\vec{Z}$$

Here, *r⃑* = (*x*, *y*, *z*) is the spatial position of the voxel to which the model is assigned, *t* is time location in the pulse sequence, *z⃑* is the unit vector in *z* direction, *G* is a time-varying imaging gradient term, and Δ*B*_ma_ is a local macroscopic fiel offset that characterizes the main fiel imperfection within the voxel. The term *C_i_* (*r⃑*) accounts for variations in the *i*^th^ compartment response due to its chemical shift. To allow for fl xible modeling of chemical shift effects (e.g., multi-peak fat spectra [32]), we represent this term by a discretized spectral model

(7) $$C_{i}=\sum_{k}m_{i,j}\Delta c_{i,k},\;\sum_{k}m_{i,k}=1$$

Here, Δ*c*_*i*, *k*_ and *m*_*i*, *k*_ are *k*^th^ spectral offset and amplitude, respectively. The last term in Eq. (6), *δB*_mi_, is a local microscopic fiel deviation with respect to Δ*B*_ma_ introduced for stochastic modeling of $T_{2}^{*}$ decay along the lines of [5, 33, 34]. In this approach Eqs (1)(2) are solved several times for the same voxel, with a value of *δB*_mi_(*r⃑*) randomly drawn from the inverse Cauchy-Lorentz cumulative distribution as

(8) $$\delta B_{\text{mi}}(\vec{r})=\frac{1}{\lambda T_{2}^{\prime }}\tan([\pi (N(\vec{r})-0.5)])$$

where *N* (*r⃑*) is a random variable uniformly distributed in [0…1], and $1/T_{2}^{\prime }=1/T_{2}^{*}+1/T_{2}$. The macroscopic voxel signal is calculated as an average of all such signals.

### B. Design of Anatomical Objects

The generalized exchange model introduced in the previous section enables fl xible modeling of signal from a single volume element. For imaging simulations, the anatomy of interest can be represented as a collection of such elements. In MRiLab, a particular tissue type (e.g., in case of brain white/gray matter, lesions, cerebrospinal flui (CSF), etc.) is related to a given voxel in the digital object by assigning the voxela tissue-specifi configuratio of the generalized model and model parameter values. Partial voluming (PV) effect can be simulated by discretizing the object at fine levels than the image resolution targeted by the simulations. Alternative approach to simulate PV is to assign to the given voxel an aggregate model corresponding to all intra-voxel tissues.

### C. Simulation of Imaging Experiment

**Fig. 2:**
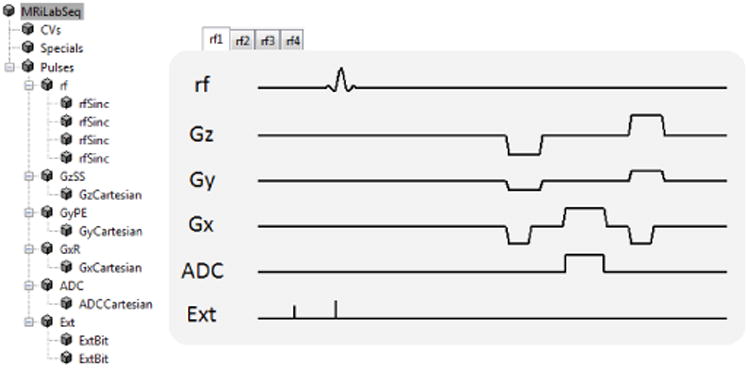
An example of a hierarchical balanced steady state precession (bSSFP) sequence tree structure with the corresponding generated waveforms. Four separate RF sources in the tree permit modeling of parallel RF transmission (for display purposes, only one RF source within one *T R* is shown). The pulse sequence is built from tunable macros which provide modularization and reusability.

In addition to tissue and anatomical models, the realistic simulations require setting up a virtual MRI system and a pulse sequence which conform to the existing physiological and technical limits of MRI scanning. MRiLab parameters specifying the scanning environment include the maps of main magnetic (B_0_), transmit, and receive fields and parameters of imaging gradients. The pulse sequence is built graphically (Fig. 2) to defin time-varying RF pulses and imaging gradients (all checked against the prescribed limits of the virtual MRI system) to obtain the desired image contrast, resolution, and acquisition trajectory. The pulse sequence can be augmented by programmable external events that can be activated at any prescribed time point to adjust the Bloch equation solution (e.g., setting transverse magnetization to zero to simulate spoiling) and to model real-time processes such as motion-induced object changes and changes in model parameters (e.g., due to contrast agent propagation, respiration-induced B0 variations, etc.).

Once the digital object, scanner environment, and pulse sequence are set up, the simulator begins by performing the solution of the multi-pool exchange ordinary differential equations (ODE) (Eqs. (1)(2)). Our approach is to utilize a discrete time solution of the Bloch equation by means of rotation and exponential scaling matrices at each time point throughout the prescribed pulse sequence [35]. Such approach was also employed in several single-component simulators [4],[28]; it does not require the use of dedicated CPU-optimized numerical ODE solvers that were engaged in Ref [5]. As the solutions for the elements in the digital object are independent of each other, the performance of such simulations benefit significantl from the remarkable parallelization capabilities of a GPU. Therefore, we utilized Compute Unifie Device Architecture (CUDA) model (Nvidia Inc, Santa Clara, CA, USA) to gain computational power sufficien for manipulation of a large spin matrix of the generalized multi-pool exchange model for a large number of the digital object voxels simultaneously. In MRiLab, GPU runtime setup is optimized based on the object size and GPU card specifications Namely, several computational blocks are created to allow maximized usage of GPU streaming multi-processers. Each block is configure to contain the maximum possible number of threads (one thread performing calculations for only one voxel) for the block's register pool of a given CUDA compilation (63 registers/thread in our case, which led to 20-65 blocks with 483-500 threads each in the simulations presented in the paper). GPU global memory is reserved to store object information and current spin status. In each step, the central processing unit (CPU) loads the GPU shared memory of each block with the next pulse sequence segment until the memory is fille or pulse sequence external event is detected. The equations are then solved for the given segment for all voxels assigned to the block's threads. Upon completion, the GPU blocks are updated with new voxels, and the process continues until ODEs are solved for all voxels. At this point, if the current pulse sequence segment ends by an external event, CPU updates GPU global memory to reflec the changes specifie by the event, and the algorithm proceeds to the next sequence segment. The cumulative signal from all the voxels forms a simulated *k*-space dataset which can be further processed to reconstruct fina images using built-in or external image reconstruction modules.

## III. Methods and Results

The frontend of MRiLab (main console, design and visualization tools) was implemented in Matlab (MathWorks Inc, Natick, MA, USA). The computational kernels were implemented in C++ and interfaced with Matlab functions. All simulations were performed on a desktop computer (Intel Xeon W3520 quad-core CPU with 12 GB DDR3 RAM and Nvidia Quadro K4200 graphic card (1344 CUDA cores, 4GB GDDR5 RAM)) running a 64-bit Windows 7 operation system. In the simulations, the number of realizations in Eq. (8) was set to 100. All experiments were performed with identically independently distributed, complex-valued Gaussian noise added to the simulated k-space data.

**Fig. 3:**
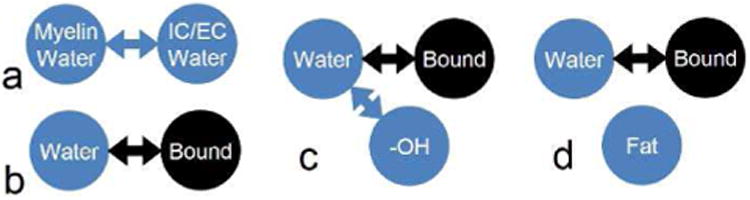
Configuration of the generalized exchange model to represent tissue response in (a) multi-component *T*_2_ relaxometry for myelin water imaging, (b) quantitative MT imaging, (c) gagCEST imaging, and (d) MT imaging in the presence of fatty tissue infiltrations

We applied the multi-pool simulator to assess several quantitative methods which either cannot be evaluated or can be evaluated only approximately by single-component MRI simulators. These simulations entailed several non-trivial configuration of the generalized exchange model (Fig. 3) described in the next sections.

### A. Multicomponent T_2_ Relaxometry

In the firs study, we evaluated the effects of simplifie modeling of multi-component *T*_2_ relaxometry on tissue microstructure characterization. Multi-component *T*_2_ relaxometry separates MRI signal into slow and fast relaxing components, which are often related to biologically important microstructural features. For example, in neural tissues, the short *T*_2_ (*T*_2,s_) signal originates from water trapped in bi-layers of myelin (the protective sheath critical for neural fibe functioning), and the long *T*_2_ (*T*_2,l_) signal corresponds to intra/extracellular (IC/EC) water [18]. The ratio of short *T*_2_ component to the total water signal, the myelin water fraction (MWF), can be used for assessment of myelin, which is a major site for pathology in a variety of disorders [36].

MWF imaging can be accurately modeled using two water proton pools connected by a diffusion-driven magnetization exchange (Fig. 3a), whose rate depends on the thickness of myelin sheath [37]. The single-component simulators can implement this model only approximately by specifying two isolated (non-exchanging) spins with different *T*_2_ values in a voxel. To illustrate the importance of multi-pool modeling implemented in our simulator, we evaluated the effect of this simplificatio on MRI signal and MWF quantification The simulations were performed using the full (Fig. 3a) and the simplifie (two water pools, no exchange) models in a cylindrical object for multiple spin echo sequence (see Appendix for sequence and model parameters).

**Fig. 4:**
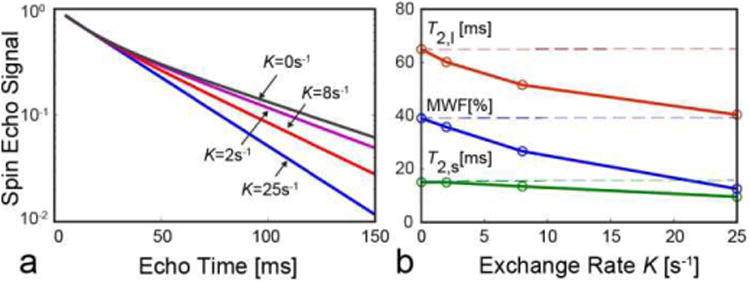
Dependence of spin echo signal (a) and apparent MWF and *T*_2_ values (b) on the exchange rate *K*. In (b), the dashed lines show true parameter values and markers correspond to *K* values from (a). The deviations of signal and parameter values grow with the exchange rate.

Figure 4a demonstrates that the spin echo signal obtained by the approximate model (isolated spins with exchange rate *K* = 0) deviates significantl from the signal obtained with consideration of inter-compartmental exchange (Fig. 3a). The deviation grows with *K*. Figure 4b demonstrates that ignoring the magnetization exchange in standard simulators adversely affects estimation of *T*_2_ components and MWF. In this simulation, the simplifie (no-exchange) model was fi to signals generated with the full model. MWF and *T*_2_ of both water pools become underestimated, with relative bias growing together with inter-compartmental exchange. The relative MWF errors of the non-exchanging model are [-8, -28, -67] % for *K* = [2, 4, 25] s^−1^, which is in agreement with the previously reported MWF errors [37]. Therefore, the use of simplifie (no exchange) model realized in standard simulators can neither represent variations in the image contrast due to variations in the exchange rate (e.g., with myelin thickness [37]) nor accurately simulate MWF mapping experiments.

### B. Quantitative MT Imaging (qMTI)

In this numerical experiment, we evaluated the ability of MRiLab to simulate quantitative MT-based assessment of tissue macromolecules with non-measurable (i.e., rapidly decaying) transverse magnetization. MT effect is observed in MR images when magnetization of macromolecular protons is selectively saturated by off-resonance RF pulses. The saturation propagates to water protons through magnetization exchange thereby causing attenuation of measurable MRI signal. Consequently, the tissue in the MT experiment can be represented as exchanging macromolecular (bound) and free (water) proton pools (Fig. 3b) [30]. The key parameter of the model, macromolecular proton fraction (MPF), is highly sensitive to many types of macromolecules including myelin and collagen, which can be affected by pathology in a variety of diseases (e.g., myelin in multiple sclerosis [38, 39], collagen in osteoarthritis [40]). Simulating the MT phenomenon requires dedicated modeling of macromolecular (bound) protons and their interaction with tissue water (Eq. (2)) which is not possible in standard simulators based on single-component models.

**Fig. 5:**
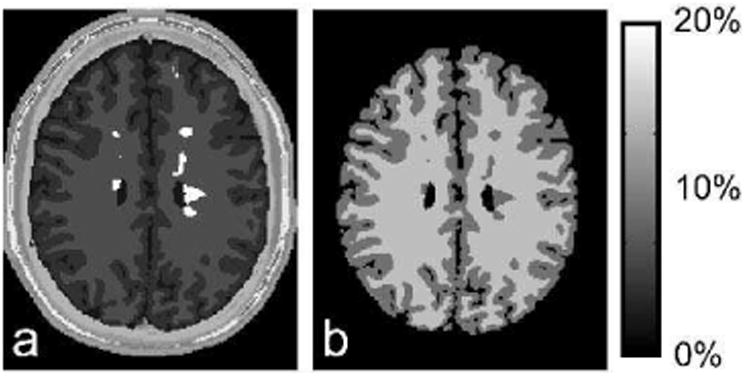
(a) Digital object used in qMTI simulation experiments. Brain parenchyma is composed of white and gray matter, and lesions. (b) Ground truth MPF.

We simulated MPF mapping using a fast qMTI protocol known as modifie cross-relaxation imaging (mCRI) [16]. The mCRI estimates MPF from a series of MT-weighted, variable fli angle (VFA) spoiled gradient echo (SPGR) images using approximate analytical expressions. The protocol also acquires a fli angle map using Actual Flip Angle (AFI) pulse sequence [41] for correction of local excitation fli angle and MT saturation power in the model fit All acquisitions were simulated at 3T for a brain template with MS lesions [42] (Fig. 5) at two resolutions, one with the acquisition matrix matching that of the digital model (200×160×60), and the other with a coarser acquisition matrix (128×96×20) to simulate PV effects (see Appendix for the full list of simulation parameters). B_1_ fiel was simulated by an MRiLab module for an eight-channel transmission coil composed of Biot-Savart linear filaments Flip angle and MPF maps were calculated fittin AFI [41] and mCRI [16] equations using in-house software.

**Fig. 6:**
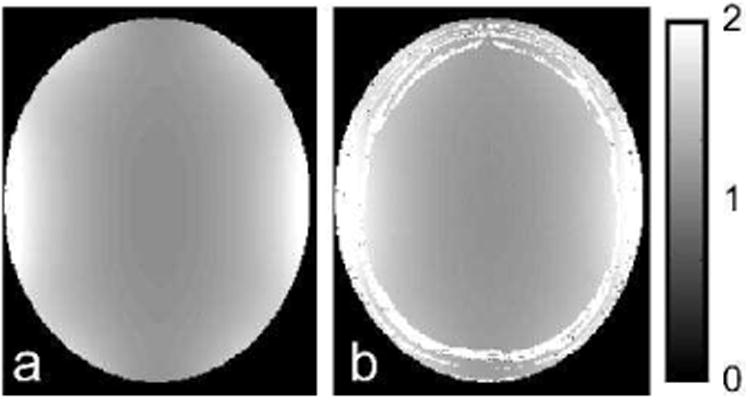
True fli angle (FA) (a) and simulated FA (b) maps. The maps are shown in units relative to the nominal (operator-prescribed) value.

**Fig. 7:**
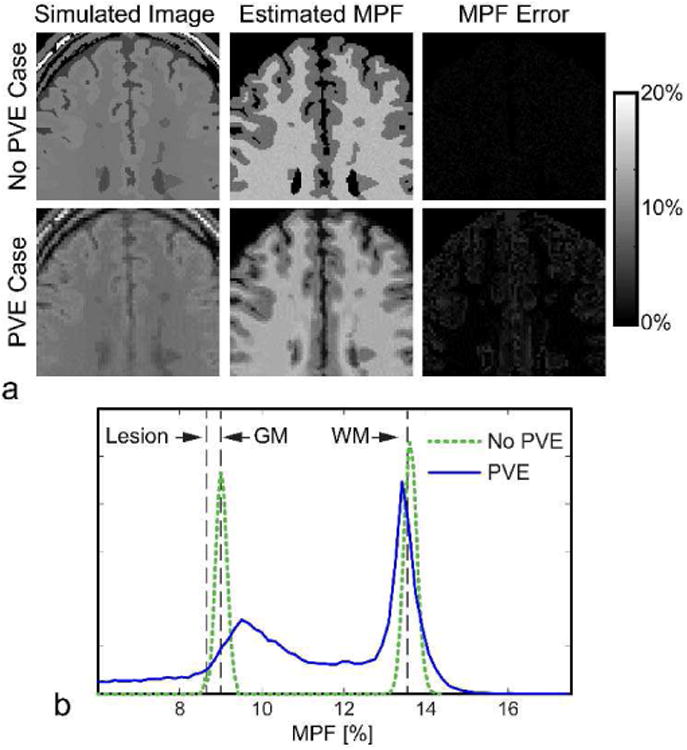
Results of simulation-based evaluation of MPF mapping. (a) Example simulated image, estimated MPF, and MPF errors. (b) MPF histograms. Vertical lines correspond to the true MPF values. Note different locations of the histogram modes for simulations with and without PV.

Figure 6 shows ground truth fli angle (FA) map, and the map estimated from MRiLab-simulated AFI sequence. The maps agree well with each other resulting in normalized root-mean-square-error = 0.9% over the brain area, which is consistent with the previously observed fli angle mapping errors due to approximations inherent to the AFI technique [41]. Figure 7 shows results of simulated MPF mapping, which provides a measure of macromolecular protons invisible with conventional MRI techniques. The macromolecular proton modeling implemented in MRiLab yielded MPF estimation highly consistent with ground truth. The values demonstrated minor biases (0.6%, 1.0%, 0.5% errors in gray matter (GM), white matter (WM), and lesions, respectively), partially due to analytical approximations used in mCRI method and propagation of the FA estimation error. Partial voluming of WM and GM cause their MPF histogram peaks (Fig. 7b) to deviate significantl from the true values. Remarkably, partial voluming between GM and CSF manifests itself as a long histogram tail in the lower MPF range, which is consistent with artificia reduction of MT-based parameters in the outer GM cortex observed experimentally [43]. The errors are also elevated in the voxels corresponding to PV between CSF and brain (MPF error image in Fig. 7a) indicating that models even more complex than two-pool MT model are required to account for partial voluming with CSF [24].

### C. Glycosaminoglycan CEST Imaging

Glycosaminoglycan CEST (gagCEST) imaging is a method to assess cartilage for the presence of glycosaminoglycan molecules [44], whose depletion is an early marker of osteoarthritis (OA). The protons in hydroxyl (-OH) groups of the glycosaminoglycan molecules are chemically shifted by +1 ppm with respect to the main water resonance. The off-resonance saturation can be applied at the shifted frequency to selectively saturate protons in OH-groups, which in turn saturate water protons through the chemical exchange. The presence of the molecules can be detected by analyzing the chemical-shift induced asymmetry of the signal (*S*) at positive (+*σ*) and negative (−*σ*) off-resonance saturation frequencies (Z-spectrum) calculated as

(9) $$\text{gagCEST}(\delta )=\frac{S(-\delta )-S(+\delta )}{S(-\delta )}\times 100\%$$

To investigate the formation of gagCEST asymmetry, we simulated in MRiLab gagCEST imaging at 7T The model configuratio consisted of three exchanging pools (Fig. 3c) representing bound protons in collagen, tissue water protons, and free protons in the hydroxyl groups (see the Appendix for simulation parameters). Additionally, we simulated the gagCEST asymmetry using an approximate model consisting of two non-exchanging free proton pools (-OH and water), which can be implemented in standard simulators.

**Fig. 8:**
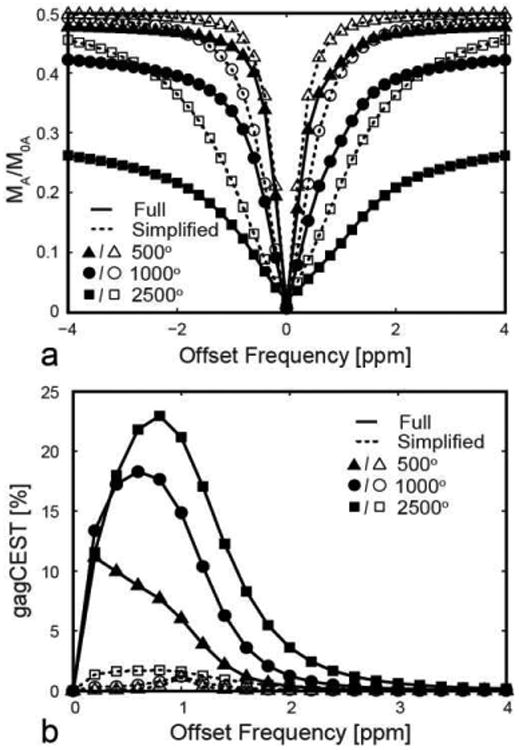
Comparison of gagCEST simulations using simplifie (two non-exchanging pools, dashed lines) and full (three exchanging pools, solid lines) models. (a) gagCEST Z-spectra and (b) the asymmetry plots simulated for simplifie and full models for several off-resonance saturation powers (*α*_CEST_ = 500°, 1000°, 2500°).

The simulations with the three pool gagCEST model (Fig. 3c) yielded Z-spectra and its asymmetry plot typical for experimental gagCEST data [44]. All spectra have slight asymmetry around 1 ppm (Fig. 8a), especially pronounced on the asymmetry plot (Fig. 8b), which signifie the presence of hydroxyl protons exchanging with the free water. The maximum value of the asymmetry is remarkably different between the models ranging from ∼1% (simplifie two-pool model) to ∼23% (full model). Simulating the phenomenon using the simplifie model is equivalent to direct detection of -OH groups, which is not feasible in vivo due to their scarcity (200-300mM) [44,45]. Full modeling of the saturation transfer in MRiLab simulates their effect on much more abundant, and hence detectable, water protons, and creates a more realistic estimation of asymmetry levels observed for -OH experimentally [44-47].

### D. MT Imaging in the Presence of Fat

In this experiment, we coupled advanced multi-pool modeling capabilities of MRiLab with a physical phantom measurements to elucidate effects of fat-water interference in macromolecular-rich tissues, which were experimentally shown to obfuscate interpretation of MT-weighted MRI signal [48]. The pure fat and water mixtures (e.g., in breast and liver tissues) can be represented in single-component simulators thanks to the absence of efficien mechanisms of magnetization exchange between fat and water protons [49]. Similarly, interactions between water and macromolecules can be evaluated using recently proposed qMTLab software [26]. However, the simultaneous presence of MT-inducing macromolecules, water, and fat makes these standard models insufficient Instead, a more advanced three-pool model comprising exchanging macromolecules and water, and non-exchanging fat (Fig. 3d) [49] is necessary to describe such tissues, which can be instantiated in MRiLab.

The phantoms were prepared by mixing the heated 2% agar water solution with peanut oil to yield fat fractions of 0%, 30% and 50%. The MR images were simulated for the digital objects and the pulse sequence identical to those used in real MRI experiments (see Appendix for model and pulse sequence parameters). In both real and simulated cases, magnetization transfer ratio (MTR) was calculated for each echo time from images with (*MT*_on_) and without (*MT*_off_) MT saturation:

(10) $$\text{MTR}=\frac{\left({\text{MT}}_{\text{off}}-{\text{MT}}_{\text{on}}\right)}{{\text{MT}}_{\text{off}}}\times 100\%$$

Because of low MTR-to-noise ratio in phantom data (ranging from 0.95 to 4.2), *MT*_on_ and *MT*_off_ were pre-processed prior to MTR calculation using local polynomial filte [50]. The agreement between experiment and simulated results was determined in Bland-Altman analysis (±1.96 standard deviation of the mean difference was as limit of agreement). The bias between simulations and experiment was examined using the one-sample *t*-test for the differences between paired measurements with the significanc level define as *p* < 0.05.

**Fig. 9:**
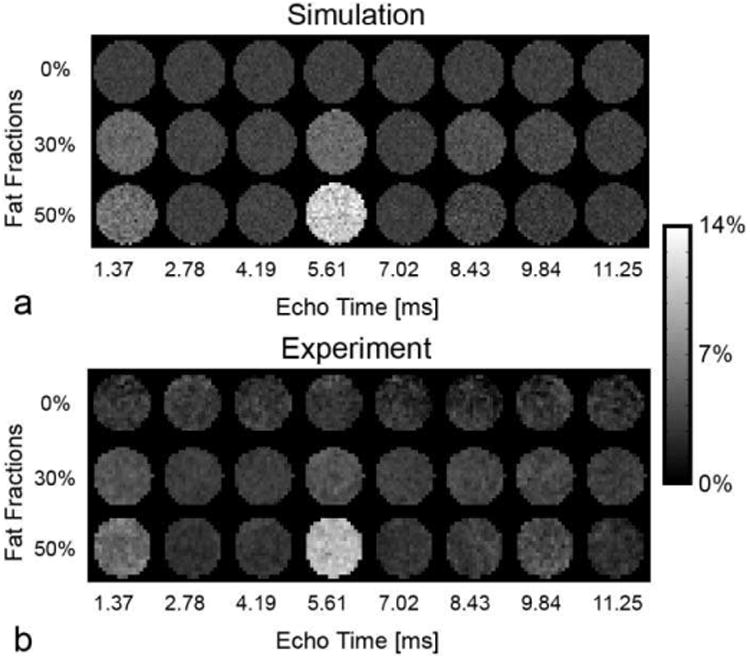
MTR in fat+agar phantoms at different echo times calculated by simulation (a) and measured at 3.0T (b). Note significan variability of MTR with fat fraction and echo time.

**Fig. 10:**
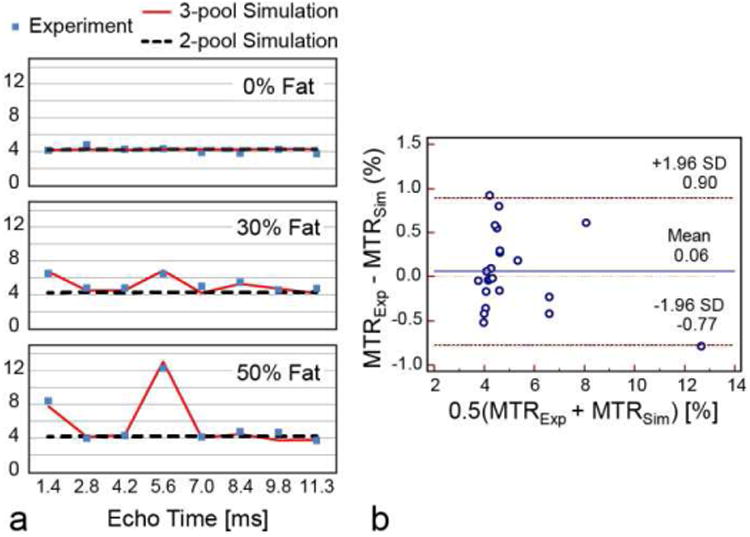
(a) Simulated and measured ROI-averaged MTR values agree well with each other for a range of echo times and fat fraction. (b) The Bland-Altman plot for experimental and simulated MTR values.

Figure 9 shows measured and MRiLab-simulated MTR images of agar/water/fat phantoms. Figure 10a compares corresponding ROI-averaged MTR values. Simulations with the standard two-pool MT model (i.e., with 0% fat) yield stable signal across different echo times. Experimental data reveal that the presence of fat leads to a fluctuatin MTR, which cannot be explained by the standard model (Fig. 10a) highlighting difficultie in interpretation of MT-based macromolecular markers in tissues containing fat. At the same time, the three-pool model describes well the echo-time and fat-content dependent superposition of chemically shifted fat signal with MT-attenuated water signal. The three-pool simulations agree well with the experimental observations as revealed by narrow limits of agreements (−0.77% +0.90%) and non-significan bias (0.06 ± 0.43%, *p* = 0.68) between simulation and experiment (Fig. 10b). This agreement supports validity of the three-pool MT model with fat component (Fig. 3d) for interpretation of MT-weighted signal in tissues containing a mixture of fat, water, and macromolecules [49], which cannot be otherwise accomplished by a standard two-pool MT model.

### E. Computational Performance

We firs compared speeds of GPU-based and standard CPU-based multi-threaded parallel computations in MRiLab (bSSFP scanning of a brain phantom [42] with *TR*/*TE* = 6/3ms, *α* = 15°, acquisition matrix 200×160, 30 slices, single water component). The CPU code was written in C using OpenMP technique for multi-threaded execution, and two matrix processing libraries (IPP (Intel Inc, Santa Clara, CA, USA) and Framewave (Advanced Micro Devices Inc, Sunnyvale, CA, USA) for accelerated CPU-based matrix operations. Next, we compared computational times for simulating this pulse sequence with all model configuration described in studies 1-4 and several acquisition matrix sizes. All simulations were repeated 10 times and the average simulation time was recorded.

Table 1 compares computational times of CPU-based and GPU-based calculations in MRiLab. GPU-based parallelization resulted in a nearly 200-fold improvement in computational speed compared to standard single threaded CPU computations, with the improved speed not achievable with standard multi-threading available on a regular personal computer. Table 2 shows computational times for different models. The computational times increased with the model complexity from qMTI and multi-component *T*_2_ relaxometry (two pools, one exchange pathway) to MT/fat imaging (three pools, one exchange pathway) to gagCEST imaging (three pools, two exchange pathways).

## IV. Discussion

There exist several key distinctions between proposed MRi-Lab and existing MRI simulators. In MRiLab, the generalized multi-pool exchange model is combined with a computational engine designed for large scale, high fidelit simulations of MRI processes (please see the online user manual at http://mrilab.sourceforge.net/ for the full description of MRi-Lab functionality). The ability to simulate actual imaging sets MRiLab apart from software that evaluate multi-pool systems in a single-voxel regime (e.g., two-pool MT modeling software [26]), and makes MRiLab particularly appealing for evaluation of conventional and quantitative methods in realistic imaging conditions. Next, while the single-component imaging simulators may imitate multi-component modeling by accommodating spins of several types per imaging voxel, such approach does not take into account exchange processes and cannot represent macromolecular tissue content. On the other hand, MRiLab numerically solves Bloch equations for the general tissue model that encompasses multiple exchanging water and macromolecular pools and thus avoids these limitations. Similar to other comprehensive MRI simulators [4, 5], MRiLab resolves the computational challenges associated with large-scale 3D simulations and numerical solution of Bloch equations using parallel computing. However, instead of engaging expensive computer cluster hardware, it relies on relatively cheap personal computer-based GPU, which, to the best of our knowledge, was previously used only in a single-component MRI simulator [28]. Despite lower computational power of a GPU core compared to a CPU core, the ample number of cores in GPU and high amenability of MRI simulations to parallelization allow achieving accelerations on two orders of magnitude relative to a single-thread CPU implementation (Table 1). Our experiments demonstrated that GPU acceleration is also capable of tackling the extra computational complexity associated with the incorporation of fl xible multi-pool models into the MRiLab simulation pipeline (Table 2).

We demonstrated utility of multi-component MRiLab simulations with several quantitative MRI experiments that are not assessable by standard single-component MRI simulators. In addition to studying the limitations of simplifie modeling with single-component simulators for gagCEST and multi-component *T*_2_ mapping, we applied MRiLab to confir the accuracy of modifie cross-relaxation imaging (mCRI), an efficien qMTI technique for mapping myelin in neural tissues and collagen in cartilage, which was previously evaluated only experimentally [16]. Furthermore, multi-component MRiLab simulations can provide explanations for variations of MT contrast with echo time and fat fraction in the presence of tissue fat, which is not possible using single component models. Previously, these effects were observed in liver MT imaging [48]. Successful comparison of MRiLab predictions with actual MRI measurements in phantoms with known fat-tissue composition confirme the necessity of the three pool model (Fig. 3) for simulation of MT imaging in the presence of fat.

The utility of fast multi-pool simulations for quantitative imaging extends well beyond the example applications provided in this manuscript. MRI simulations with the multi-pool models can be valuable in many stages of development of quantitative techniques, including preliminary concept evaluation, evaluation of expected imaging performance, and assessment of the dependence of the accuracy and precision of model outputs on imaging and reconstruction parameters. Furthermore, the existing MRiLab functionality allows the simulator to be used for an even wider variety of simulations tasks. For example, external events implemented in MRiLab can be used to perform a dynamic update of the model parameters. This mechanism can be utilized to simulate tissues undergoing dynamic changes; for example, in dynamic contrast-enhanced imaging, which uses two-pool contrast kinetics modeling to quantify perfusion/permeability [23].

From software design perspective, MRiLab builds on the ideas of pipeline processing [11] and modularization [51], which makes MRiLab simulation structure fl xible and extensible. The extensibility is particularly facilitated by the use of Extensible Markup Language to store simulation information, to register new modules, and to organize communication between predefine macros and external programs. The latter may be straightforwardly applied to create a communicating pipeline for incorporating functions of external programs. The combination of high computational efficien y, extensibility, and open-source concept makes MRiLab an appealing platform for further expansion by existing or future models of MRI processes.

Similar to any existing MRI simulator, MRiLab may be limited by simplifie description of physical processes that are problematic to model numerically using currently available computational power. For example, direct numerical simulation of diffusion effects based on random-walk modeling during the pulse sequence evolution may require exhaustive computational power. In addition, in all shown experiments, spoiling of the transverse magnetization was achieved through an external event zeroing the transverse magnetization, which may not be sufficien to model real experiments in which no special arrangements are made in the pulse sequence design to achieve complete spoiling [52]. In these cases, a more accurate approach to model the spoiling gradient effects on the intra-voxel transverse magnetization is through fine discretization of the digital object grid. This approach, however, may reach memory and computational feasibility limits, as MRiLab simulations are primarily restricted by the available memory size and the resources each thread can assess (i.e., shared memory and registers). However, rapid advances of new GPU-based methods (e.g. multiple-GPU and GPU cluster) [53] and faster and more powerful GPU devices could be used in the future to further improve the time-efficien y of the MRI simulation and to extend the simulation complexity in MRiLab to address these and other complex simulation problems. Finally, as studies in this manuscript were tested under CUDA 2.0, the backward compatibility to earlier versions of CUDA model is likely to require source code modification Future development will include providing the support for freely available programming platforms such as NumPy/SciPy [54] to broaden the availability of MRiLab to the scientifi community.

## V. Conclusion

In this paper, we presented a comprehensive, high-performance, open-source MRI simulation tool capable of realistic simulations of the whole MRI experiment with fl xible representation of tissues by multi-pool exchange models. We demonstrated the feasibility of such full-scale MRI simulations on a regular personal computer equipped with relatively inexpensive GPU hardware. The MRiLab simulation environment can serve as a f exible, readily available, expandable platform for convenient customizing virtual MRI experiments to streamline the development of new MRI methods. This simulator may be particularly useful for accelerated development and accurate evaluation of new MRI approaches designed to assess tissue composition and microstructure in a quantitative fashion.

## Figures and Tables

**Table I:** MRILAB Simulation Times for GPU and Multi-Threading CPU Parallelization

CPU (Intel XeonW3520)				GPU (Quadro K4200)
1 thread	2 threads	4 threads	8 threads	
83402 sec	42005sec	20700 sec	10412 sec	419 sec

**Table II:** Simulation Times for Several Configurations of the Generalized Model and *K*-Space Matrix Sizes

Model Type	k-space Matrix Size		

	64×64	128×128	256×256
Single Pool	102 sec	293 sec	921 sec
qMTI	171 sec	478 sec	1496 sec
Multi-Component T2	183 sec	614 sec	1612 sec
MT + fat	235 sec	651 sec	2142 sec
gagCEST	241 sec	699 sec	2214 sec

## Appendix

### A. Multicomponent T_2_ Mapping

Images at several echo times were obtained simulating multiple spin echoes for the model in Fig. 3b. The simulations were performed in cylindrical objects each assigned an individual exchange rate value. The model (Fig. 3a) and pulse sequence parameters were identical to ones used in [37]. *Model parameters: T*_2,s_/*T*_2,1_ = 15/65ms, MWF = 39%, myelin water exchange rate *K* = [0, 2, 8, 25] s^−1^. *Multiple spin echo sequence: TR* = 6s, *TE* = [5, 10, 15, …, 150] ms. *Other details:* object size 100×100×30 (number of voxels 300000), *k*-space-matrix size 60×60, pulse sequence time steps 241560, total simulation time 176 sec.

### B. Quantitative MT Imaging

The two-pool MT model (Fig. 3b) and pulse sequence parameters were similar to the ones reported in [16]. All datasets were simulated in axial plane with fiel of view = 20 × 16cm. *Model parameters:* Gray matter: *T*_1,*w*_ = 1.4 s, *T*_2,w_ = 100 ms,*T*_1,b_ = 1s, *T*_2,b_ = 10.21 *μ*s, *K*_w,b_ = 1.57 s^−1^, MPF = 8.9%. White matter: *T*_1,w_ = 1s, *T*_2,w_ = 70ms, *T*_1,b_ = 1s, *T*_2,b_ = 9.84*μ*s, *K*_w,b_ =2.70*s*^−1^, MPF = 13.6% MS lesions: *T*_1,w_ = 1.3 s, *T*_2,w_ = 30 ms, *T*_1,b_ = 1 s, *T*_2,b_ = 9.84 *μ*s, *K*_w,b_ = 2.70 s^−1^, MPF = 8.5% *MT-SPGR sequence: TR/TE* = 37/3.5 ms, excitation fli angle *α* = 15°, a 18ms Fermi MT pulse, all combinations of **Ω** = 2.5, 10, 18, 26 kHz and *α*_MT_ = 850°, 1400°. Same sequence was used to simulate variable fli angle data with *α* = 6°, 15°, 35°, 50°, **Ω** = 250 kHz. *AFI sequence: TR*_1_/*TR*_2_ /*TE* = 37/185/2.3 ms, *α =* 55°. *Other details (cases without/with partial voluming effect (no-PVE/PVE):* object size 200×160×60 (number of voxels 1920000), 3D simulations with *k*-space-matrix sizes 200×160×60 (no-PVE) and 128×96×20 (PVE), pulse sequence time steps 65484900 (no-PVE) and 11524500 (PVE), total simulation times 120080 sec (no-PVE) and 23264 sec (PVE).

### C. gagCEST Imaging

The gagCEST model (Fig. 3c) and pulse sequence parameters were similar to the ones reported in previous cartilage imaging studies [44, 55]. *Model parameters:* Two-pool MT *T*_1,w_ = 1s, *T*_2,w_ = 35ms, *T*_1,b_ = 1s, *T*_2_,_b_ = 7*μ*s, *K*_w,b_ = 8s^−1^, MPF=15% [40, 56]. Glycosaminoglycan hydroxyl (-OH) pool: proton fraction 1% chemical shift *δ* = + 1.0ppm, *T*_1,-OH_ = 1s, *T*_2,-OH_ = 90ms, *K*_w,-OH_ = 12s^−1^. *MT-SPGR sequence: TR/TE=* 200/8ms, *α* = 10°, a 100ms Hanning-windowed Gaussian MT pulse, saturation fli angles *α*_CEST_ = [500°, 1000°, 2500°], **Ω** varying linearly in range [-4.0…4.0] ppm. The spectra were normalized to signals without saturation (**Ω** = 250 kHz). *Other details:* object size 100× 100×30 (number of voxels 300000), 2D simulations with *k*-space-matrix size 60×60, pulse sequence time steps 10606200, total simulation time 4800 sec.

### D. MT Imaging in the Presence of Fat

Imaging was performed on a 3T MRI scanner (MR750, GE Healthcare, Waukesha, WI) using multi-echo MT-SPGR sequence. The parameters for model in Fig. 3d were selected according to the used fat fractions and previously reported parameters for fat [32] and 2% agar [14]. *Model parameters:* Agar: *T*_1,w_ = 2.38 s, *T*_2,w_ = 56.4 ms, *T*_1,b_ = 1 s, *T*_2,b_ = 15.3 *μ*s, *K*_w,b_ = 0.734 s^−1^, MPF = 0.66% Fat: *T*_1,f_ = 280 ms, *T*_2,f_ = 55ms, 6-peak fat spectra, peak fractions/chemical shifts [8.7/−3.1, 69.3/−2.75, 12.8/−2.11, 0.4/−1.57, 3.9/−0.32, 4.8/0.49] %/ppm. *MT-SPGR sequence: TR* = 40 ms, *TE* = [1.37, 2.78, 4.19, 5.61, 7.02, 8.43, 9.84, 11.25] ms, excitation angle *α* = 13°, 18ms Fermi MT pulse, *α*_MT_ = 1000°, **Ω** = 2.5 kHz and 250 kHz. *Other details:* object size 195×161×10 (number of voxels 313950), 2D simulation with *k*-space-matrix size 100×80, pulse sequence time steps 45500, total simulation time 340 sec.
